# The Impact of Ammoniation Treatment on the Chemical Composition and In Vitro Digestibility of Rice Straw in Chinese Holsteins

**DOI:** 10.3390/ani10101854

**Published:** 2020-10-12

**Authors:** Yulin Ma, Xu Chen, Muhammad Zahoor Khan, Jianxin Xiao, Shuai Liu, Jingjun Wang, Zhiyuan He, Congcong Li, Zhijun Cao

**Affiliations:** 1 State Key Laboratory of Animal Nutrition, Beijing Engineering Technology Research Center of Raw Milk Quality and Safety Control, College of Animal Science and Technology, China Agricultural University, Beijing 100193, China; ma18810318038@163.com (Y.M.); SY20193040671@cau.edu.cn (X.C.); zahoorkhattak91@163.com (M.Z.K.); dairyxiao@gmail.com (J.X.); liushuaicau@cau.edu.cn (S.L.); wangjingjun@cau.edu.cn (J.W.); hezhiyuan@cau.edu.cn (Z.H.); 2 State Key Laboratory of Animal Nutrition, Institute of Animal Sciences, Chinese Academy of Agricultural Sciences, Beijing 100193, China; congcongli@caas.cn

**Keywords:** ammoniation treatment, rice straw, corn steep liquor, in vitro digestibility

## Abstract

**Simple Summary:**

Rice straw has many essential uses as a byproduct of agriculture. As a feed source, due to low digestibility, low crude protein and minerals contents, the pretreatment of rice straw is required before use in ruminant feeding. To enhance the nutritive value of rice straw, different methods are practiced. Among them, treatment with ammoniation might be effective regarding the rice straw intake, enhancement of straw digestibility and crude protein levels, which are essential for enhancing the productive ability of dairy cattle. In the current study, we experimentally proved the efficiency of ammoniation treatment to enhance the different feed value parameters (dry matter digestibility, neutral detergent fiber, crude protein, gas production, acetic acid, butyric acid, and total volatile fatty acid) of rice straw.

**Abstract:**

The current study was conducted to explore the ammoniation treatment effects on the chemical composition and in vitro digestibility of rice straw in Chinese Holsteins. For this purpose, rice straw was stored in polyethylene bags (35 × 25 cm, 350 g per bag) including (i) no additives (RS); (ii) 5% urea (5U, dry matter (DM) basis); (iii) 9% corn steep liquor + 5% urea (9C5U, DM basis); (iv) 9C2.5U; and (v) 9C2.5U + 3% molasses (9C2.5U3M, DM basis). The air-dry matter of the mixture was kept at the same level at 55% for all treatments. Fifteen bags (5 treatments × 3 repeats) were prepared and stored at ambient temperature (25 ± 3 °C). The chemical composition and in vitro digestibility were measured at day 60 after storage. Our analysis revealed that all the four ammoniation treatments improved the in vitro DM and neutral detergent fiber (IVNDFD) digestibility. In addition, all the four ammoniation treatments significantly (*P* < 0.001) increased the levels of crude protein (CP), gas production (GP), acetic acid (AA), butyric acid (BA) and total volatile fatty acid (TVFA) contents of the rice straw and decreased the neutral detergent fiber (NDF) and acid detergent fiber (ADF) of the rice straw compared to the control. Within four treated groups, the 9C5U treatment was most effective. Finally, we concluded that ammoniation treatments increased the nutritive value of rice straw. In addition the 9C5U treatment could be an effective ammoniation treatment for the better utilization of rice straw.

## 1. Introduction

China is one of the largest producers in agricultural production, and the annual crop straw production was approximately 810 million tons in 2013 [1], of which 190 million tons was rice straw [2]. Rice straw is the major source of biomass yield, and rice grain is distributed throughout the globe because of its importance as a kind of food crop for more than half of the world’s population [3]. The increasing consumption of grain leads to a large amount of straw residue, including rice straw with low utilization. The low utilization rate of rice straw is mainly because of its low nutritive value, which is commonly considered as a medium-low quality feed in dairy cattle rearing [4,5]. Therefore, concerns in improving the nutritional quality and utilization rate of rice straw by ruminants are stayed increasing.

So far, various preprocessing methods have been explored in practice, such as physical, chemical and biological treatment to enhance the nutritional quality of straw [6]; ammoniation is the most key chemical technique among them [7]. Ammoniation has been developed to break lingo-cellulosic bonds in crop residues, thereby enhance the nutritional quality of rice straw [8]. Urea is a commonly used chemical product in ammoniation treatment [9]. However, it has been reported that urea treatment only retains 30% to 35% of the NH_3_ released during the treatment [10]. Feeding cows the urea treated rice straw, the retained nitrogen would rapidly liberate in the rumen and cause nutrient losses [11]. The release rate of urea in the rumen is too fast, and the available nitrogen is much higher than the requirement of energy and nitrogen balance. At present, many researchers are focusing on reducing the release rate of nitrogen in the rumen to achieve the effect of energy and nitrogen balance [12]. To tackle this problem, some researchers utilized H_2_SO_4_ or HCl to fix NH_3_ [8]; however, fixing the NH_3_ in straw with acids was costly and dangerous. The addition of urea can increase the total nitrogen content in the feed, but with the release of ammonia, the pH value increases, resulting in the fermentative quality of the silage declined [13].

Corn steep liquor may solve the problem of escaping NH_3_ of urea treated rice straw. It is a kind of by-products of processed corn starch, rich in carbohydrates and can significantly increase the crude protein (CP) content of rice straw [14]. Corn steep liquor has an ability to fix NH_3_ because of its acidic characteristic [15], which increased the CP content in the rice straw. Nisa et al. studied the effect of supplementation of 0%, 3%, 6% and 9% dry matter (DM basis) of corn steep liquor to wheat straw treated with 5 kg urea (50% moisture level) [16]. A higher retained nitrogen content and improved digestibility of DM and neutral detergent fiber (NDF) were found in the diet containing corn steep liquor of Buffalo bulls. In addition, as the concentration of corn steep liquor increased, the rumen volatile fatty acid (VFA) and acetic acid (AA) concentrations increased, while the highest VFA concentrations were found with the addition of 9% corn steep liquor [15]. As a feed flavor additive, molasses could enhance the palatability [17] and increase the feed intake of ruminants. Furthermore, it has been reported that molasses improve the quality of silage fermentation [18]. However, most of the previous studies have not considered the combined effect of urea, corn steep liquor and molasses on the nutritive value and digestibility of rice straw.

Therefore, the aim of the study was to explore the effects of different ammoniation treatment methods on the nutritional quality parameters (chemical composition, in vitro digestibility, in vitro gas production and in vitro rumen fermentation performance) of rice straw. In addition, the current study evaluated the process to provide a scientific foundation for the utilization of rice straw as feed.

## 2. Materials and Methods

### 2.1. Raw Materials and Silage Preparation

The nutrient content of rice straw (reaped in the rural farmland of Gushi County Xinyang city, Henan Province, China) and corn steep liquor (purchased from Henan Yuyao New Medicine Co. LTD, Xinyang, China) analyzed by wet chemistry has summarized in Table 1. The total nitrogen (N) content and ammonia volatilization inhibition rate of Urea (Purchased from Henan Hand-in-Hand Fertilizer Co. LTD) was ≥46% and ≥5%, respectively.

The rice straw was chopped to about 3–5 cm by a grinder. Then a given amount of rice straw (about 350 g, based on the breathing fermentation bag) were treated with (i) no additive (RS); (ii) 5% urea (5U, dry matter (DM) basis); (iii) 9% corn steep liquor + 5% urea (9C5U, DM basis); (iv) 9% corn steep liquor + 2.5% urea (9C2.5U, DM basis); and (v) 9% corn steep liquor + 2.5% urea + 3% molasses (9C2.5U3M, DM basis). For each treatment, the amounts of urea, corn steep liquor and water were calculated in advance and weighed, materials were separately added distilled water to adjust the moisture content to 45% and then vacuum-packed in a 35 cm × 25 cm breathing fermentation bag (VAY8828, Yongkang Jiegu Food Machinery Co. LTD, Yongkang, China). Fifteen bags (5 treatments × 3 repeats) were made and stored at ambient temperature (25 ± 3 °C). Three bags for each treatment were opened after 60 days; all samples were oven-dried at 65 °C for 48 h and ground in a hammer mill to pass 1-mm sieve for the determination of chemical compositions and in vitro digestibility.

### 2.2. Chemical Analysis

The dry matter (DM), crude fiber (CF), crude protein (CP) and crude ash content (Ash) of the samples were determined according to the method described by AOAC (1984) [19]. The neutral detergent fiber (NDF) and acid detergent fibre (ADF) were analysed through a method established by Van Soest et al. [20]. The content of NDF and ADF was measured by using the ANKOM 2000i automatic fiber analyzer (Beijing Anke Borui Technology Co. Ltd., Beijing, China). The determination of acid detergent lignin (ADL) concentration was conducted according to the method described in the National standard (GB/T 6433-2006, GB/T 20805-2006). 

### 2.3. Rumen Fluids Collection

Three Holstein cows weighing approximately 600 kg/cow, lactation number 2, day of lactation was 152 ± 14 and milk yield 38 ± 3 kg per day, equipped with permanent rumen fistulas were used as rumen fluid donor animals. The cows were fed three times a day (07:30, 14:30, and 18:30), with free access to water. The basic diet and nutrient levels have been provided in Table 2.

### 2.4. In Vitro Rumen Incubation

In the current study, the automated trace gas recording system (AGRS) type microbial fermentation gas production system was used to detect the cumulative gas production (GP) [21]. Rumen fluid from three Holstein cows was collected two hours after feeding in the morning and placed in a 39 °C pre-temperature vacuum flask, which was immediately transported to the laboratory. The rumen fluids of the three cows were mixed in equal proportions, squeezed through four layers of mussels before in vitro inoculation, it was placed in a CO2 bath at 39 °C.

AGRS was used to record total gas production. Each bottle (6 replicates/samples) was filled with 0.5 g of samples, 25 mL stratified rumen fluid and 50 mL medium (pH 6.85). The medium was prepared following the method developed by Menke et al. [22]. All bottles were purged with anaerobic N_2_ for 5 s, sealed with rubber plug and Hungate screw caps and individually connected with medical plastic infusion pipes to the AGRS, using the method of Zhang and Yang (2011) [23]. All the bottles were incubated at 39 °C for 48 h, and each batch culture system runs 4 bottles of stover samples for blank correction. 

After incubation for 48 h, the bottle was removed from the AGRS system. The pH value of the culture medium was immediately determined; 1.0 mL culture medium was mixed with 0.3 mL 2.5 g/L interphosphate solution at 4 °C for 30 min and centrifuged at 10,000× *g* at 4 °C for 10 min. The supernatant was stored at −20 °C for the determination of acetate acid (AA), propionic acid (PA), butyrate acid (BA) and total volatile fatty acids (TVFA). The supernatant was removed from the bottle and the residual feed was dried at 65 °C to constant weight for determination of DM and NDF. In vitro disappearance of DM (IVDMD) and NDF (IVNDFD) was calculated as a difference between the initial culture of DM, NDF, residual DM and NDF corrected by blanks. 

### 2.5. Computation

Cumulative gas production has been recorded using the AGRS-Ⅲmicrobial fermentation gas production system and obtained according to the exponential function model proposed by Groot et al. [24];

GP_t_ = A/[1 + (C/t)^B^]

The GPt is the total gas production (mL/g DM) at time t; A is the theoretical maximum gas production (mL/g DM) of the fermentation of substrate at this gas production rate; B is the curve inflection point parameter during fermentation; C is the time (h) to reach the maximum gas production rate 1/2; t is the gas production time (h).

$$AGPR\;=\;\frac{A\times B}{4\times C}$$

In the formula: AGPR stands for the gas production rate when the maximum gas production is reached 1/2.

### 2.6. Statistical Analysis

All the data were analyzed using the IBM SPSS Statistics24. One-way ANOVA analysis was performed to examine the effect of ammoniation treatment on the nutritional content of rice stover and gas production in vitro. Duncan multiple comparison method was carried out to compare the differences between the means; *P* < 0.05 was used to show the significance levels. 

## 3. Results

### 3.1. The Effect of Ammoniation Treatment on the Chemical Composition of Rice Straw

Compared to the RS control treatment, all four ammoniation treatments increased the CP level of rice straw. However, the CP level with treatments 9C5U and 9C2.5U was significantly (*p* < 0.001) higher than the 5U and 9C2.5U3M (Table 3). The 5U and 9C2.5U treatments had significantly (*p* < 0.001) lowest and the highest level of Ash, respectively. Ash content was medium with the 9C5U and 9C2.5U3M treatments and was significantly (*p* < 0.001) higher than the RS treatment. The 9C5U and 9C2.5U3M treatments had a significantly (*p* < 0.001) lower level of NDF compared to the other treatments. The 9C5U and 9C2.5U treatments had a significantly (*p* < 0.001) lower level of ADF than the RS and 5U treatments. The 9C5U treatments had a significantly (*p* = 0.032) lower level of ADL compared to 5U and 9C2.5U treatments, however no difference was found between 5U and 9C2.5U treatments. No difference (*p* = 0.930) was documented among all treated groups in terms of DM.

### 3.2. Effect of Ammoniation Treatment on In Vitro Digestibility of Rice Straw

Results of IVDMD and IVNDFD are presented in Table 4. The IVDMD and IVNDFD of the 9C5U treatment were significantly greater than the other treatments. Consequently, the RS treatment had the lowest IVDMD and IVNDFD (*p* < 0.001), while no significant difference (*p* > 0.05) was found among the 5U, 9C2.5U and 9C2.5U3M treatments. 

### 3.3. Effect of Ammoniation Treatment on Gas Production of Rice Straw In Vitro

**Figure 1:**
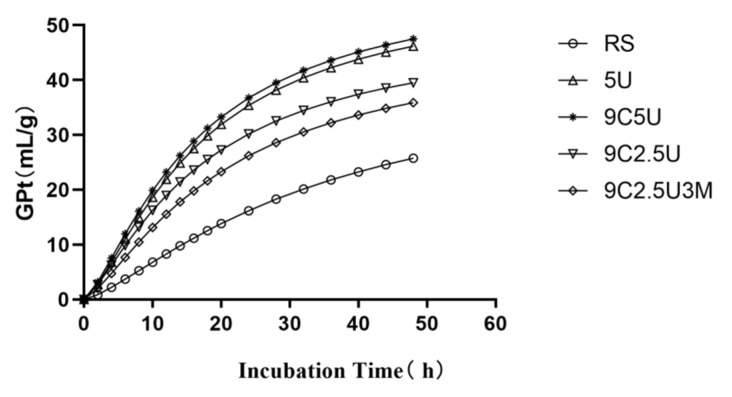
Cumulative gas production curve at 48 h at four ammonia treatments (RS: no additive control; 5U: addition of 5% urea; 9C5U: 9% corn steep liquor + 5% urea; 9C2.5U: 9% corn steep liquor + 2.5% urea; 9C2.5U3M: 9% corn steep liquor + 2.5% urea + 3% molasses).

The changes in the gas production during in vitro culture process are shown in Figure 1. With the extension of the in vitro culture time, the gas production of each group gradually increased, while the fastest (the slope was the largest) gas production was recorded in response to treatments 5U and 9C5U.

The gas production kinetic characteristics of rice straw after 60 days of anaerobic storage are showed in Table 5. All four ammoniation treatments increased the GP_48_ of rice straw with the 9C5U and 5U treatments having considerably (*p* < 0.001) higher GP_48_ than the 9C2.5U and 9C2.5U3M treatments. The 5U and 9C2.5U treatments had the significantly highest level (*p* < 0.001) of A, respectively. Having the intermediate level of A, the 9C2.5U treatment was higher than the RS treatment (*p* < 0.001). The 9C5U, 5U and 9C2.5U treatments had a notably (*p* < 0.001) lower level of C compared to the other treatments. Having an intermediate level of C, the 9C2.5U3M treatment was lower than the RS treatment (*p* < 0.001). The 5U and 9C5U treatments had a significantly (*p* < 0.001) higher level of the AGPR than the RS, 9C2.5U and 9C2.5U3M treatments. No difference (*p* = 0.270) was noticed between the treatments in terms of the B.

### 3.4. Effect of Ammoniation on In Vitro Rumen Fermentation Parameters of Rice Straw

As shown in Table 6, all four ammoniation treatments increased the AA content of rice straw with the 9C5U and 5U treatments having considerably (*p* < 0.001) higher content of AA than the 9C2.5U3M treatments (*p* < 0.001); consistently, the AA content in 9C2.5U treatment was significantly higher than 9C2.5U3M. Ammoniation treatments had a notably (*p* < 0.001) higher content of BA compared to the RS treatments, and the 5U treatments had a considerably (*p* < 0.001) higher content of BA than 9C5U, 9C2.5U and 9C2.5U3M treatments. The 9C5U, 9C2.5U3M and 5U treatments had a significantly (*p* < 0.001) higher content of PA than the 9C2.5 and RS treatments. The 9C5U and 5U treatments had a notably (*p* < 0.001) higher content of TVFA than the RS and 9C2.5U3M treatments, and the TVFA of 9C5U and 9C2.5U treatments were significantly higher than RS (*p* < 0.05); there was no significant difference between the 9C5U and 9C2.5U treatments (*p* > 0.05). There was no significant difference in pH value between the groups (*p* = 0.06).

### 3.5. Correlations among the Chemical Composition, Gas Production, Rumen Fermentation Parameters and In Vitro Degradability of Rice Straw

**Figure 2:**
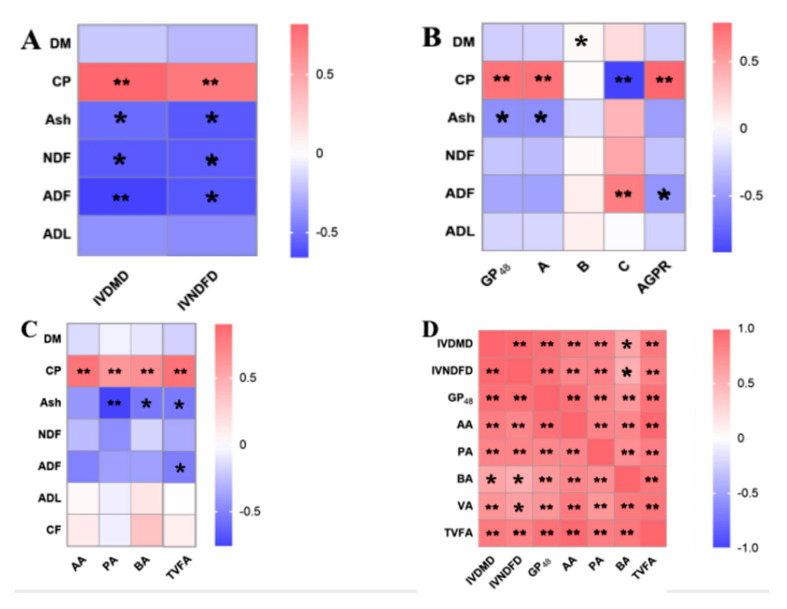
Correlations among the chemical composition, gas production, rumen fermentation parameters and in vitro degradability of rice straw. (**A**) Correlation between the chemical composition and in vitro digestibility of rice straw; (**B**) correlation between the chemical composition and gas production kinetic parameters; (**C**) correlation between the chemical composition and rumen fermentation parameters; (**D**) correlation among the in vitro digestibility, gas production and rumen fermentation parameters of rice straw. AA stands for acetic acid; PA: propionic acid; BA: butyric acid; TVFA: total volatile acid; DM: dry matter; CP: crude protein; NDF: neutral detergent fiber; ADF: acid detergent fiber; ADL: acid detergent lignin; IVDMD: in vitro dry matter degradability; IVNDFD: in vitro neutral detergent fiber degradability; GP: total gas production (mL/g DM) at time t; AGPR: The gas production rate when the maximum gas production is reached 1/2. * showed that the *p* < 0.05; **, indicates that *p* < 0.001. Blue represents a negative correlation and red represents a positive correlation.

The correlations among the chemical composition, gas production, rumen fermentation parameters and in vitro degradability of rice straw are shown in Figure 2. In this study, the increase in IVDMD and IVNDFD of rice straw was mainly due to the increase in CP content (*p* < 0.001) and decreased in Ash, NDF (*p* < 0.05) and ADF content (*p* < 0.001) (Figure 2A). The increase in GP_48_, A and AGPR of rice straw was mainly due to the increase in CP (*p* < 0.001). The Ash content was negatively correlated with GP_48_ and A of rice straw (*p* < 0.05). The ADF content was negatively correlated with C (*p* < 0.001) and AGPR (*p* < 0.05) (Figure 2B). The increase in AA, PA, BA and TVFA of rice straw was mainly due to high CP content (*p* < 0.001). The Ash content was negatively correlated with PA (*p* < 0.001), BA and TVFA (*p* < 0.05) of rice straw. The ADF content was negatively correlated with TVFA (*p* < 0.05) (Figure 2C). There was a positive correlation between IVDMD, IVNDFD, GP_48_ and TVFA (*p* < 0.001) (Figure 2D).

## 4. Discussion

Straw is a common source of feed for ruminants, but its utilization efficiency is low due to the low CP content with only 3~5% of DM and high NDF and ADF [25]. In the present study, no difference was found in the DM of rice straw for all groups, which showed that the ammonia treatment has little effect on the DM content. Furthermore, the nitrogen content of feed affects the efficiency of rumen microbial protein synthesis, and low nitrogen utilization ability will cause economic losses and environmental pollution. Our current findings revealed that 9C5U and 9C2.5U improved the CP levels of rice straw from 5.8% to approximately 11.0% after 60 days of anaerobic storage. Similar to our results, a study has shown that the nitrogen content increased in rice straw by the acidification of corn steep liquor [26]. Furthermore, with the addition of molasses, the nitrogen retention rate in rice straw was reduced, which might be due to the molasses and urea reaction [18]. The addition of corn steep liquor could increase the nitrogen retention rate in the rice straw which is why the CP content in 9C5U and 9C2.5U is higher than 5U treatment. The main reason for this might be due to the losses of ammonia in 5U treatment. Generally, a higher CP level is beneficial for higher DM digestibility [27]. This is noteworthy that in the present study, the corn steep liquor treatment improved the CP content of rice straw. Corn steep liquor promotes the proliferation of microorganisms by providing excellent carbon and nitrogen substrates metabolism, thereby increased the synthesis of functional enzymes and bacterial proteins and improved the CP content of rice straw [28]. In general, another reason for the low utilization rate of rice straw is the high content of NDF and ADF, which decreases the digestibility in ruminants. The 9C5U treatment decreased NDF and ADF of rice straw from 65.41% to 60.02% and 54.64% to 47.84% after 60 days of anaerobic storage. This showed that ammoniation treatment caused the breakdown of the complex structure of rice straw lignocellulose and dissolved a part of cellulose and hemicellulose [29]. Consistently, the ammoniation treatment ensures the sufficient nitrogen supply of microbes, which resulted in the higher degrading activity of the microbes and the resulting effects.

DM and NDF digestibility are the key characteristics that stand for the intake and production potential of forages in ruminant feeding. It was noticed in our findings that all the treatments improved IVDMD and IVNDFD of the rice straw after 60 days of anaerobic storage. Ammoniation weakens the hydrogen bonding inside the rice straw; result in the expansion of fiber molecules and the ester bond or ether bond. Bacteria are the main settlers in the rumen and therefore make the most significant contribution to the fermentation, degradation and digestion of feed. It was documented in previous findings that the barley straw fibers treated with urea cultured in the rumen of animals have the most bacteria attached and the change of rumen environment significantly increased the degradability of rumen bacteria [30,31]. The 9C5U treatment was most effective which may be due to the addition of the highest quantity of urea and corn steep liquor. The level of IVDMD and IVNDFD in 9C2.5U and 9C2.5U3M treated group was lower than the 9C5U treated group, the less quantity of the urea addition in 9C2.5U and 9C2.5U3M group might be the possible reason for these differences. However, the IVDMD and IVNDFD of the 5U group were lower than that of the 9C5U group because of no corn steep liquor addition, which makes the rumen microorganism obtain less nutrients and shorts the rumen fermentation [32]. There was no statistical difference among 5U, 9C2.5U and 9C2.5U3M. Moreover, in the corn steep liquor-added group, the IVDMD and IVNDFD were higher than the control group. We expected that differences might be due to the rapid and simultaneous release of corn steep liquor and urea to a certain extent, which increased the growth efficiency and the number of microorganisms [14,33]. On the other hand, the DM degradation rate can reflect the digestibility of the feed to a certain extent. It is related to the nutritional composition of the feed. Generally, a higher degree of synchronization of energy and nitrogen release increases the digestibility of feed nutrients in part by affecting the bacterial community, metabolism and enzyme activity of ammonia assimilation in vitro fermentation [34].

During in vitro fermentation, the digestibility of feed organic matter is significantly correlated with gas production. The higher the digestibility of organic matter, the fermentation activity of microorganisms in the rumen increases, the gas production rate is accelerated, and the gas production is further increased [35]. In the present study, the GP_48_ and AGPR of each ammoniation treated groups were higher than that of the control group, indicating that the ammoniation can increase the soluble carbohydrate of rice straw and increase the gas production rate and the gas production. It was noticed that chemical treatment changed the rice straw cell wall structure and the solubility of cellulose and hemicellulose is improved [36], which can be explained by significant differences in NDF and ADF content. The results of in vitro studies further indicate that the higher the urea content, the greater gas production in vitro, which is consistent with the results of the Senthilkumar’s findings [37]. The GP_48_ in the 9C5U and 5U groups were the highest and was consistent with their low NDF and ADF content. In the 9C2.5U and 9C2.5U3M groups, the urea content was reduced and the NDF and ADF content was higher, which may lead to lower gas production in vitro, But they are better than the control group.

The pH value of the treatment groups in this study was not significantly different within the normal range from 6.75 to 6.82, which was consistent with the report by Bath et al. [38]. This shows that the effect of different compound ammoniation methods on the rumen pH was not obvious in this study. The AA, PA and BA were increased in the compound ammoniation treatment groups compared with the control group, which may be due to the enhancement in the digestibility of rice straw [39], making it more degradable, and increased VFA production. Among them, the contents of AA, PA and TVFA in the 9C5U and 5U treatment groups were the highest, which was consistent with their highest gas production in vitro. The 9C5U, 5U, 9C2.5U and 9C2.5U3M treatments had a higher PA level than the control. A previous report had documented that the PA production was positively correlated with nitrogen deposition, and the higher CP content in these treatments than control also explains this phenomenon [40]. Besides CP, the microbial supply and activity were also enhanced by ammoniation treatment which is also one of the leading factors responsible for the higher level of PA.

Ammoniation treatment degraded the side chains of esters and glycosides leading to structural modification of lignin, cellulose swelling, cellulose decrystallization, and hemicellulose solvation and increased the degrading activity of the microbes [41], and this diversification might affect the NDF and ADF of rice straw. In the current study, the NDF and ADF content of rice straw and IVDMD and IVNDFD also showed a negative correlation. In addition, the CP content in the straw was positively correlated with digestibility, gas production and rumen VFA content [42], which are in line with our current findings.

It was previously reported that rice straw treated with urea (50 g of urea per kg DM straw) was used to replace elephant grass to feed lactating cows. It was found that the use of urea-treated rice straw increased the replacement amount of elephant grass until 75% of the roughage. Furthermore, rice straw had increased the milk fat content, and has no effect on milk yield and other milk composition parameters. Feeding rice straw treated with urea could partially replace the elephant grass in the lactating cow’s diet during the dry season, which reduced the cost of roughage [43]. In addition, feeding rice straw treated with urea enhanced the rumen ecology, rumen fermentation efficiency and nutrient digestibility of ruminants [44]. Studies have shown that rice straw treated with 20 g/kg urea + 20 g/kg calcium hydroxide can increase nitrogen retention and microbial protein synthesis [45].

## 5. Conclusions

Based on our findings, we concluded that all ammoniation treatments effectively increased CP content, reduced NDF content, and increased the IVDMD and IVNDFD digestibility, as well as VFA concentration and GP_48_ of rice straw in vitro. However, the 9C5U treatment was most effective by enhancing the nutritional compositions and in vitro digestibility of rice straw. Thus, here we recommended that 9% corn steep liquor and 5% urea treatment (9C5U) could be considered to maximize the nutritional composition and in vitro digestibility of rice straw. Finally, our study provided the basis for the better utilization of rice straw as an animal feed source. In addition, the results of the most promising variants should be approved with a higher number of replicates.

## Figures and Tables

**Table 1:** Contents of chemical components of raw materials (DM basis).

Item	Rice Straw	Corn Steep Liquor
DM (%)	92.3	41.5
EE (%)	1.5	-
CP (%)	4.9	43.0
NDF (%)	70.6	-
ADF (%)	41.2	-
Ash (%)	16.9	17.0

DM: dry matter; CP: crude protein; EE: ether extract; NDF: neutral detergent fiber; ADF: acid detergent fiber.

**Table 2:** Basic diet composition and nutrient value (DM basis %).

Item	Content	Nutrient Levels ^4^	Content
Oat hay	5.6	NEL (MJ/Kg)	6.50
Alfalfa hay	11.5	CP	18.01
Alfalfa silage	8.3	EE	2.96
Corn silage	24.5	NDF	32.33
Steam-flaked corn	13.7	ADF	21.80
Corn	5.0	Ca	0.50
Soybean meal	8.4	P	0.35
Soybean hull	5.2		
Corn DDGS	4.4		
Sprayed corn skin	3.3		
Cottonseed meal	3.3		
Molasses	2.9		
Berg + Schmidt ^1^	0.5		
XP XPC	0.3		
Premix ^2^	2.4		
NaHPO _3_	0.4		
OPTIGEN ^3^	0.3		

^1^ The main components of Berg + Schmidt feed additive are crude fat ≥ 99.5%; water and impurities ≤ 0.5%; acid value, mg KOH/g ≦ 2; peroxide value, meq/kg ≤ 5. ^2^ Each kilogram of premix contains 11.0% calcium, 0.7 mg/kg copper, 1.1 mg/kg zinc, 2.2 mg/kg manganese, 76 mg/kg iodine, 5.5 mg/kg selenium, 29 mg/kg cobalt, and vitamin A 116,500 IU/kg, vitamin D3 57,000 IU/kg, vitamin E 750 IU/kg. ^3^ The main component of OPTIGEN is the relative content of crude protein (converted from non-protein nitrogen) ≥250%, nitrogen ≥ 40%, total arsenic ≤15 mg/kg, lead ≤ 40 mg/kg, and moisture ≤ 10%. ^4^ Except that the net energy for lactation content is calculated according to the raw materials of the Chinese Dairy Cattle Feeding Standard (NY/T34-2004), the rest of the nutrients were measured values.

**Table 3:** Effect of ammoniation treatment on the chemical composition of rice straw (%) after 60 days of storage.

Item	DM	CP	Ash	NDF	ADF	ADL
RS	95.96 ± 0.90	5.79 ^c^ ± 0.15	13.91 ^b^ ± 0.08	65.41 ^a^ ± 0.78	54.64 ^a^ ± 1.05	1.30 ^ab^ ± 0.07
5U	95.87 ± 0.01	9.27 ^b^ ± 0.06	12.94 ^e^ ± 0.04	65.30 ^a^ ± 0.82	53.19 ^b^ ± 0.13	1.35 ^a^ ± 0.08
9C5U	95.69 ± 0.06	11.38 ^a^ ± 0.57	13.32 ^c^ ± 0.02	62.02 ^c^ ± 0.01	47.84 ^d^ ± 0.16	1.22 ^b^ ± 0.00
9C2.5U	95.86 ± 0.06	11.02 ^a^ ± 0.09	14.20 ^a^ ± 0.07	64.07 ^b^ ± 0.02	48.90 ^cd^ ± 0.00	1.36 ^a^ ± 0.02
9C2.5U3M	95.96 ± 0.17	9.51 ^b^ ± 0.01	13.18 ^d^ ± 0.01	62.37 ^c^ ± 0.20	49.72 ^c^ ± 0.98	1.30 ^ab^ ± 0.01
*p*-value	0.930	<0.001	<0.001	<0.001	<0.001	0.032

Different superscript letters a, b, c, d, and e indicates significantly different values (*p* < 0.05) column-wise, and the same or no letters indicate insignificant differences (*p* > 0.05). RS: no additive control, 5U: add 5% urea, 9C5U: 9% Corn steep liquor + 5% urea, 9C2.5U: 9% Corn steep liquor + 2.5% urea, 9C2.5U3M: 9% Corn steep liquor + 2.5% urea + 3% Molasses. DM: dry matter (the dry matter content is calculated based on air-drying the sample); CP: crude protein; NDF: neutral detergent fiber; ADF: acid detergent fiber; ADL: acid detergent lignin; The CP, Ash, NDF, ADF and ADL content are all calculated based on absolute dry matter.

**Table 4:** Effect of ammoniation treatment on the digestibility of DM and NDF of rice straw in vitro after 60 days of storage.

Item	IVDMD	IVNDFD
RS	52.49 ^c^ ± 0.71	41.73 ^c^ ± 1.37
5U	63.49 ^b^ ± 0.08	55.15 ^b^ ± 2.95
9C5U	69.43 ^a^ ± 0.46	62.85 ^a^ ± 1.15
9C2.5U	59.96 ^b^ ± 0.69	48.35 ^b^ ± 0.47
9C2.5U3M	59.83 ^b^ ± 0.08	50.39 ^b^ ± 0.21
*p*-value	<0.001	<0.001

Different superscript letters a, b and c indicates significantly different values (*p* < 0.05) column-wise, and the same or no letters indicate insignificant differences (*p* > 0.05). RS: no additive control; 5U: add 5% urea; 9C5U: 9% corn steep liquor + 5% urea; 9C2.5U: 9% corn steep liquor + 2.5% urea; 9C2.5U3M: 9% corn steep liquor + 2.5% urea + 3% molasses.

**Table 5:** Effect of ammoniation treatment on gas production of rice straw in vitro.

Item	Aerodynamic Parameters				
	GP_48_ (mL/g DM)	A (mL/g DM)	B	C/h	AGPR (mL/g DM)
RS	31.33 ^c^ ± 0.25	41.66 ^c^ ± 0.09	1.35 ± 0.04	33.55 ^a^ ± 1.12	0.42 ^d^ ± 0.04
5U	46.00 ^a^ ± 0.25	56.89 ^a^ ± 1.05	1.39 ± 0.11	16.76 ^c^ ± 0.05	1.19 ^a^ ± 0.21
9C5U	47.13 ^a^ ± 0.79	58.41 ^a^ ± 0.54	1.36 ± 0.12	16.27 ^c^ ± 0.05	1.25 ^a^ ± 0.02
9C2.5U	39.07 ^b^ ± 0.88	49.83 ^b^ ± 0.81	1.32 ± 0.27	17.33 ^c^ ± 0.48	0.95 ^b^ ± 0.03
9C2.5U3M	36.44 ^b^ ± 0.79	47.34 ^bc^ ± 0.24	1.34 ± 0.06	20.43 ^b^ ± 0.54	0.78 ^c^ ± 0.04
*p*-value	<0.001	<0.001	0.270	<0.001	<0.001

Different superscript letters a, b, c and d indicate significantly different values (*p* < 0.05) row-wise, and the same or no letters indicate insignificant differences (*p* > 0.05). RS: no additive control; 5U: addition of 5% urea; 9C5U: 9% corn steep liquor + 5% urea; 9C2.5U: 9% corn steep liquor + 2.5% urea; 9C2.5U3M: 9% corn steep liquor + 2.5% urea + 3% molasses; GP48 = gas production in 48 h; A = theoretical maximum gas production; B = the curve inflection point parameter during fermentation; C = the maximum gas production rate ½; AGPR = the maximum gas production is reached 1/2.

**Table 6:** Effect of ammoniation on in vitro rumen fermentation parameters of rice straw.

Item	Acetate (mmol/L)	Propionic (mmol/L)	Butyrate (mmol/L)	TVFA (mmol/L)	pH
RS	41.48 ^d^ ± 1.54	18.62 ^c^ ± 5.01	5.35 ^c^ ± 0.79	67.07 ^d^ ± 6.22	6.80 ± 0.03
5U	54.44 ^a^ ± 2.38	22.59 ^a^ ± 2.23	7.54 ^a^ ± 1.22	87.76 ^a^ ± 2.97	6.75 ± 0.04
9C5U	53.56 ^ab^ ± 2.70	22.32 ^a^ ± 2.27	6.61 ^b^ ± 1.33	85.53 ^ab^ ± 4.15	6.79 ± 0.06
9C2.5U	51.45 ^b^ ± 1.66	20.26 ^b^ ± 1.56	6.91 ^b^ ± 0.68	81.78 ^bc^ ± 3.24	6.82 ± 0.10
9C2.5U3M	47.81 ^c^ ± 0.90	21.85 ^a^ ± 3.30	6.96 ^b^ ± 0.97	79.65 ^c^ ± 2.78	6.77 ± 0.03
*p*-value	<0.001	<0.001	<0.001	<0.001	0.06

Different superscript letters a, b, c, d and e indicate significantly different values (*p* < 0.05) row-wise, and the same or no letters indicate insignificant differences (*p* > 0.05). RS: no additive control; 5U: addition of 5% urea; 9C5U: 9% corn steep liquor + 5% urea; 9C2.5U: 9% corn steep liquor + 2.5% urea; 9C2.5U3M: 9% corn steep liquor + 2.5% urea + 3% molasses; TVFA: total volatile fatty acid.
